# Multi-Targeted Molecular Effects of *Hibiscus sabdariffa* Polyphenols: An Opportunity for a Global Approach to Obesity

**DOI:** 10.3390/nu9080907

**Published:** 2017-08-20

**Authors:** María Herranz-López, Mariló Olivares-Vicente, José Antonio Encinar, Enrique Barrajón-Catalán, Antonio Segura-Carretero, Jorge Joven, Vicente Micol

**Affiliations:** 1 Instituto de Biología Molecular y Celular (IBMC), Universidad Miguel Hernández (UMH), Edificio Torregaitán, Elche 03202, Spain; mherranz@umh.es (M.H.-L.); maria.olivares@umh.es (M.O.-V.); jant.encinar@umh.es (J.A.E.); e.barrajon@umh.es (E.B.-C.); 2 Department of Analytical Chemistry, University of Granada, Avda. Fuentenueva s/n, Granada 18071, Spain; ansegura@ugr.es; 3 Research and Development of Functional Food Centre (CIDAF), PTS Granada, Avda. del Conocimiento s/n., Edificio BioRegión, Granada 18016, Spain; 4 Unitat de Recerca Biomèdica, Hospital Universitari Sant Joan, Institut d'Investigació Sanitària Pere Virgili, Universitat Rovira i Virgili, Reus 43201, Spain; jjoven@grupsagessa.com; 5 CIBER: CB12/03/30038, Fisiopatología de la Obesidad y la Nutrición, CIBERobn, Instituto de Salud Carlos III (ISCIII), Palma de Mallorca 07122, Spain

**Keywords:** antioxidants, dietary supplementation, obesity, epigenetics, metabolic stress, polyphenols, metabolites, virtual screening, *Hibiscus sabdariffa*

## Abstract

Improper diet can alter gene expression by breaking the energy balance equation and changing metabolic and oxidative stress biomarkers, which can result in the development of obesity-related metabolic disorders. The pleiotropic effects of dietary plant polyphenols are capable of counteracting by modulating different key molecular targets at the cell, as well as through epigenetic modifications. *Hibiscus sabdariffa* (HS)-derived polyphenols are known to ameliorate various obesity-related conditions. Recent evidence leads to propose the complex nature of the underlying mechanism of action. This multi-targeted mechanism includes the regulation of energy metabolism, oxidative stress and inflammatory pathways, transcription factors, hormones and peptides, digestive enzymes, as well as epigenetic modifications. This article reviews the accumulated evidence on the multiple anti-obesity effects of HS polyphenols in cell and animal models, as well as in humans, and its putative molecular targets. In silico studies reveal the capacity of several HS polyphenols to act as putative ligands for different digestive and metabolic enzymes, which may also deserve further attention. Therefore, a global approach including integrated and networked omics techniques, virtual screening and epigenetic analysis is necessary to fully understand the molecular mechanisms of HS polyphenols and metabolites involved, as well as their possible implications in the design of safe and effective polyphenolic formulations for obesity.

## 1. Introduction

Diet-induced obesity and a sedentary lifestyle are consistent with the appearance of several metabolic dysfunctions leading to metabolic syndrome (MetS). MetS is a clustering of cardio-metabolic risk factors, including abdominal obesity, insulin resistance, dyslipidemia and hypertension [1,2]. Although considerable progress has been made in understanding the molecular mechanisms underlying obesity, in many cases its treatment results in failure [3]. In this respect, caloric restriction and physical activity are the primary approaches prescribed to improve an individual´s metabolism. However, in the majority of cases, this is very difficult to maintain for a prolonged period, as it implicates important changes in the individual´s lifestyle [4,5]. Likewise, current anti-obesity pharmacological treatments are in many cases inefficient, and often present important side effects when taken for a prolonged period [6,7,8]. For this reason, many researchers and clinicians have drawn their attention towards more traditional methods to correct the obesity-induced energy imbalance. Accordingly, the use of metabolic drugs or natural dietary products, including polyphenolic xenohormetins, are some of the most interesting and promising strategies in forthcoming studies addressing obesity [9,10]. Exploring the effects of polyphenols on specific cellular pathways and diseases may turn the preventive use of natural agents into a dietary intervention in the treatment of chronic metabolic diseases. The multifactorial nature of polyphenols, a plausible consequence of their molecular diversity, offers a new opportunity to address obesity [11,12,13,14,15,16]. To this end, the main objective of this review is to elucidate the putative molecular targets of *Hibiscus sabdariffa* (HS) L. (Malvaceae) polyphenols and provide an overview of their role in the prevention of chronic diseases such as obesity.

In this review, we have collected all the relevant evidence regarding the possible benefits of polyphenolic extracts derived from HS calyces on the management of obesity-related pathologies [17,18,19,20,21,22]. These studies include the use of cell and animal obesity models, as well as human clinical trials. Furthermore, the complete characterization of this plant extract, its effective dosage, synergistic effects, bioavailability, in silico approach on selected molecular targets and localization of bioactive metabolites in tissues have been reviewed. The aim of this review is to provide a comprehensive examination clarifying whether the proper use of HS polyphenols may present an opportunity to improve the control or prevention of obesity-related diseases through the modulation of key proteins involved in oxidative stress and inflammation and, consequently, in the regulation of metabolic and bioenergetics pathways. 

In this respect, recent data have shown that AMP-activated protein kinase (AMPK) and peroxisome proliferator-activated receptors (PPAR), among others, may be possible molecular targets for HS polyphenols, as evidenced in cellular and animal models [21,23,24]. This hypothesis suggests that the active manipulation of energy sensors and effectors in obesity might be a feasible preventive therapy. Nevertheless, further research is necessary in order to delimit all the protein targets that are modulated by HS polyphenols and to elucidate their molecular mechanisms. For this purpose, metabolic profiling through “Omics” science must be used to identify the final intracellular metabolites derived from HS polyphenols that reach intracellular targets, as well as to identify endogenous metabolite biomarkers. From this perspective, validated obesity-related biomarkers could provide new diagnostic tools and establish key insights concerning the effects of natural compounds on the pathogenesis of energy-related complications.

## 2. *Hibiscus sabdariffa* Polyphenols as Xenohormetic Agents

“Let food be thy medicine and medicine be thy food” quoted by Hippocrates, and its more modern analog “an apple a day keeps the doctor away” both reflect the implicitness of food on health. Plants that traditionally served as food, fuel, water and fiber, are now being engineered as a source of natural bioactive compounds with particular attention focused on nutraceutical products and functional foods. This has in turn given rise to the concept of xenohormesis, or molecular networking between species, which tries to explain the multiple positive effects of plant-derived polyphenols on human health [25,26]. This consists in the idea that our body reacts to the signals that plants generate in periods of stress. In this sense, these biochemical signals, called xenohormetins, fulfill important metabolic and defensive functions in plants, such as protection against UV radiation and pathogen infection, nodulation, hormone transportation, as well as several other functions such as defense against herbivorism or pollination [27]. The induction of protective secondary metabolites, especially phenolics, allows plants to withstand the effect of environmental stressors, as a self-defense mechanism against external conditions [28]. Thus, this complex mechanism to protect themselves and improved throughout evolution can be in part extrapolated to humans through plant food consumption [9,29], challenging their own genetic inheritance by modifying innate responses [10].

Several thousand of these safe xenohormetic molecules have already been identified from a multitude of plant species [30]. Plants increase the synthesis of secondary metabolites upon aggressive conditions [31,32]. However, the expected amount of polyphenols in vegetables and fruits for human consumption is low and its bioavailability is limited [33]. A successful strategy to increase the intake of polyphenols would be using products derived from exotic or medicinal plants that may represent an important source of polyphenols, as well as through the proper extraction procedure and enrichment techniques [34,35,36]. Among various medicinal plants studied, HS represents a potentially optimal source of bioactive molecules for the treatment of various diseases [17,20,21,37,38,39].

**Figure 1:**
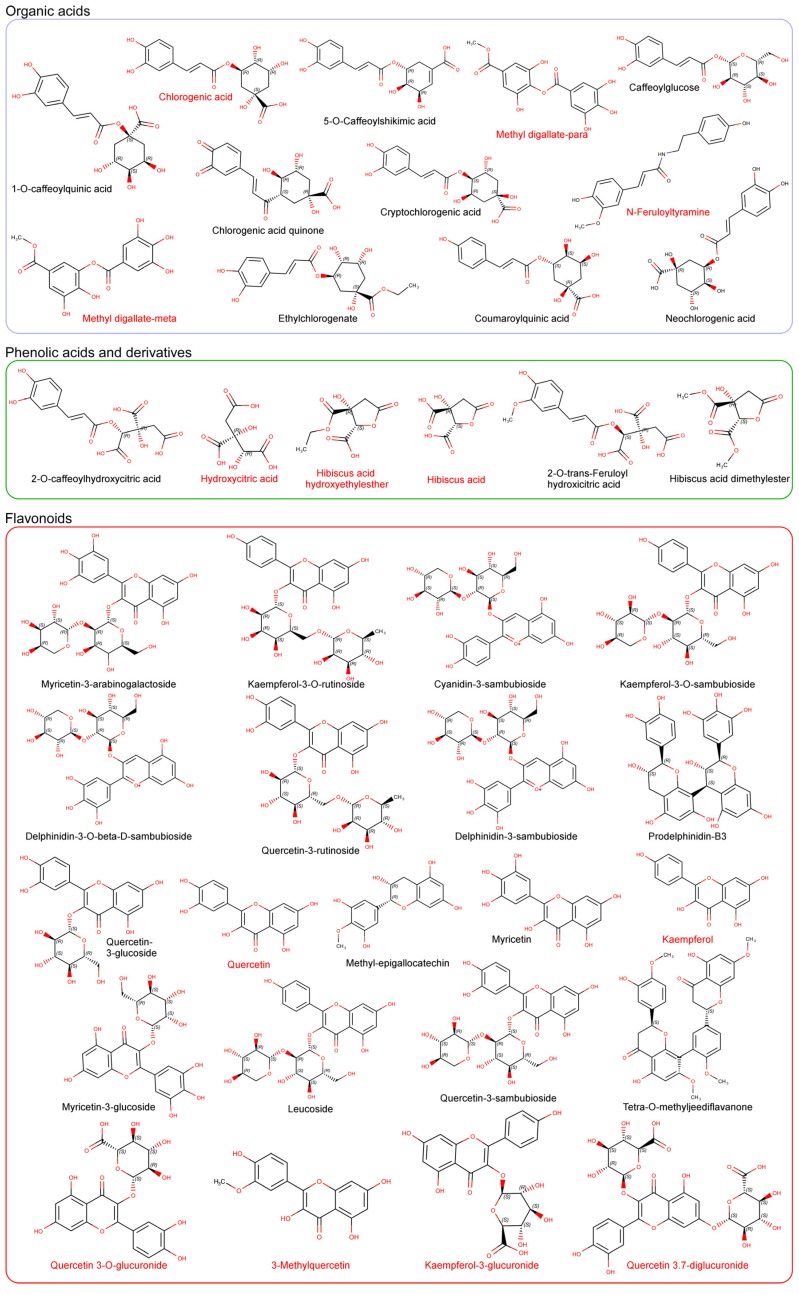
Chemical structures (explicit hydrogens have been eliminated) of the major compounds identified in *Hibiscus sabdariffa* extracts by liquid chromatography coupled to high resolution mass spectrometry. Compounds are grouped in families according to their chemical structure. The complete characterization of the extracts has been previously reported [18,20,37]. The common name of each compound is included under its structure. Compounds with their names in red indicate that these compounds have been identified as plasma metabolites in the bibliography.

In this respect, anthocyanins are one of the major polyphenolic compounds in HS aqueous extracts, and are responsible for the bright red color of the flowers. Anthocyanins in HS such as delphinidin-sambubioside (red pigment) and cyanidin-sambubioside (pink pigment) comprise the predominant anthocyanin compounds of the extract (Figure 1) and can be used as an alternative to the synthetic dyes used in the food industry [40,41]. Due to their remarkable in vitro antioxidant capability, anthocyanins have received increasing attention in the past two decades [42,43,44], and is generally accepted that anthocyanins may substantially contribute to the protective effects of HS. In fact, a correlation has been established between the antioxidant activity of HS materials and anthocyanin content, suggesting that these compounds may significantly contribute to HS´s antioxidant effect [45]. Nevertheless, extrapolation of the in vitro antioxidant properties of anthocyanins to an in vivo situation may be difficult, since these compounds are highly soluble in water and have a very short half-life, i.e., are poorly bioavailable and quite possibly not capable of reaching their molecular targets at a sufficient concentration to exert a notable effect [46]. Therefore, this suggests that the biological effect of HS extracts is not exclusively due to these antioxidant compounds. On the other hand, many studies have indicated that polyphenols present a wide variety of effects, supporting the fact that these compounds reach multiple molecular targets and exhibit molecular promiscuity [47,48], which might also endorse the xenohormesis hypothesis. In fact, HS polyphenols have recently been proposed as modulators of gene expression [21] and may be involved in several pathways related to chronic inflammation and energy metabolism [17,23,49]. Consequently, it seems necessary to discern whether the health benefits attributed to HS polyphenols are mainly due to their antioxidant effect or to their ability to modulate the activity of different target proteins, possibly through a synergic mechanism.

## 3. Characterization and Synergy of *Hibiscus sabdariffa* Bioactive Compounds

HS herbal tea has been traditionally consumed by various cultures. In the abovementioned research studies, HS was generally prepared as a standardized aqueous extract from their dried calyces, although other leaf extractions have also been used [39]. Complex plant-derived mixtures such as herbal teas suppose an important challenge for analytical procedures, although the chromatographic profile of HS aqueous extracts have been studied in detail [50]. In an attempt to identify the major bioactive compounds, several studies have focused on the chemical characterization and quantitation of extracts from HS calyces by high-performance liquid chromatography coupled to high resolution mass spectrometry (HPLC-MS) [18,20,37]. These studies revealed that anthocyanins together with organic acids are two of the most abundant groups identified in the aqueous extract [18] (Figure 1). However, phenolic acid derivatives, flavonol derivatives and phenylpropanoids have also been identified and quantified in the extract (Figure 1). HS calyces are rich in polysaccharides and soluble fiber, mainly pectins, arabinans and arabinogalactans of low molecular mass [51], but it is proposed that these compounds do not contribute to HS bioactivity [20]. HS calyces also contain a high concentration of ascorbic, arachidic, citric, stearic, and malic acids [52], making its aqueous extract quite acidic (pH = 2.8), which is ideal for maintaining polyphenolic stability. In addition, other biomolecules are present in the aqueous extracts at a low percentage, mainly unidentified proteins and peptides [53]. Therefore, such a complex plant-derived mixture may represent an opportunity for the study of potential synergistic effects on different pharmacological targets.

Plants have developed various strategies to modulate biological processes through molecular promiscuity or polypharmacologic effects, which act as multi-target drugs [54]. Due to their nature, plant bioactive compounds could have modulated their diversity throughout evolution to act as ligands of different molecular targets in animal cells. This would be responsible for the enhancement of therapies through possible synergistic interactions with multiple targets. This particular feature of plant compounds could become an opportunity to design suitable and novel approaches for diseases with complex pathogenic mechanisms [36], such as obesity-related complications.

Several studies have postulated on the putative synergistic effect of mixed botanical compounds or extracts to explain their beneficial effects [41,55,56]. Nevertheless, only a few studies have demonstrated the increased efficiency of HS extracts with respect to its isolated compounds, suggesting a therapeutic advantage for the whole extract in the treatment of complex disorders [20]. Nevertheless, it must be noted that the synergistic effect of botanical products is difficult to prove. Synergistic pharmacological interactions between compounds or drugs must be demonstrated by using specific experimental and mathematical approaches, such as the calculation of the combination index, the fractional inhibitory concentration index or isobologram construction [10]. However, this is not always easy to approach in the case of very complex matrices [57], since multiple combinations are implicated, and in many cases the polyphenolic mixtures are not fully characterized [10,58].

Plant mixtures may also exhibit an inverted U-shaped dose-effect curve of purification level vs. bioactivity, i.e., bioactivity increases up to a certain point in which the increase of the purity leads to the loss of some important compound, with the concomitant drop in bioactivity. Therefore, bioassay-guided isolation processes usually fail to reveal the major active compounds of a complex mixture. An alternative to this problem could consist in testing the whole extract and its fractions via metabolomics-guided isolation [59]. The integration of high content screening with non-targeted metabolomics could provide an additional strategy to recognize the metabolites responsible for the biological effects and facilitate the demonstration of the putative synergy in complex herbal mixtures.

## 4. Bioavailability, Tissue Distribution and Cellular Metabolites Derived from *Hibiscus sabdariffa* Bioactive Compounds

Despite the considerable efforts to characterize the HS extract and the abundance of known potential bioactive compounds, the identification of the key components and their potential targets is still largely unknown. Understanding the pharmacokinetics of compounds derived from HS extracts is essential, since their absorption, metabolism and distribution might determine the mode of action and molecular targets involved in its bioactivity. Regardless, despite the abundance of anthocyanins in HS and their attributed beneficial effects on health, several studies have revealed that these compounds present low absorption and bioavailability in vivo [60]. Therefore, it is possible that other, less abundant compounds present in HS extracts or other not yet unidentified metabolites may be responsible for the observed beneficial effects. In this respect, a bioavailability study carried out in rats revealed a total of seventeen compounds in rat plasma samples, of which quercetin and kaempferol glucuronides were the most predominant one [19]. In fact, recent studies have revealed a higher bioavailability of flavonol derivatives derived from HS compared with other alternative sources [61], with an approximate plasma concentration of 5 µM after administration in rats [19]. In this vein, the metabolite quercetin-3-glucuronide (Figure 1) was detected in hepatic and immune cells in the liver and in the intestinal mucosa of hyperlipidemic mice fed with a polyphenol-enriched extract of HS, concomitantly with an improved steatohepatitis [21]. The permeability of the polyphenolic extracts in Caco-2 intestinal human cells, a model of human intestinal absorption, has also been reported [50,62]. The data from these studies revealed a low level of permeability of the complex polyphenolic mixture through the gut barrier, suggesting that the presence of high concentrations of several polyphenols in the extract could lead to a saturation of the specific transport mechanism. In these studies, only a few compounds (*N*-feruloyltyramine and quercetin (Figure 1), among others, were able to pass through the gut barrier model. Finally, a recent study in hypertrophied 3T3-L1 adipocytes revealed the cellular absorption and metabolism of both quercetin and quercetin-3-glucuronide in adipocytes, which suggests that these flavonols might also reach other intracellular targets that contribute to their bioactivity [63]. Given all this, it could be hypothesized that the compounds responsible for the multiple effects observed with HS extracts may not be the most abundant ones, being the flavonol derivatives the possible candidates for the observed effects in animal models.

## 5. Effect of *Hibiscus sabdariffa* Compounds on Selected Digestive Enzymes

**Figure 2:**
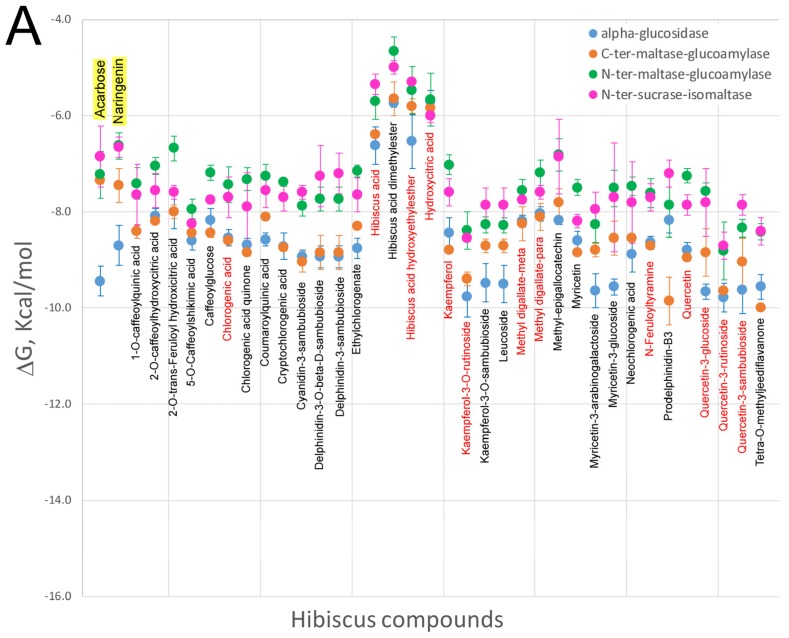

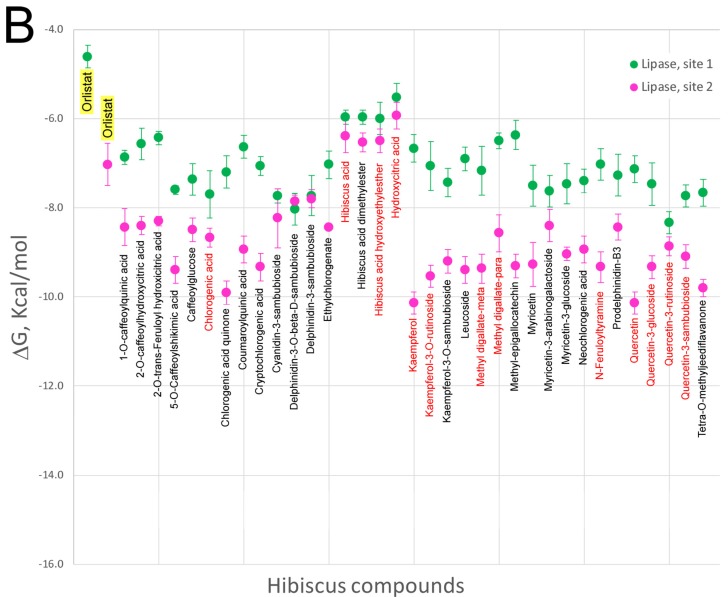
Comparison of the free energy variation (ΔG, kcal/mol) of the *Hibiscus sabdariffa* bioactive compounds and X-ray co-crystalized ligands or known modulators (first name of each panel and yellow background) against the main binding sites of the catalytic binding site of some glucosidase enzymes (panel A) and the two crystal binding sites of the triacylglycerol lipase/colipase complex (panel B) by molecular docking. The common name of each compound is included under mean and standard deviation plotted in each panel. Red colour names indicate that these compounds have been identified as plasma metabolites in the bibliography. For the virtual screening process, a chemical library containing the chemical structures to be screened was initially built, following ADMET criteria. In addition, the molecular target high resolution structures were obtained. Then, computer clusters with a high computation capability were used to compare target structures with the chemical library to find the best docking results according to DG binding values. Finally, all tested compounds were prioritized as a function of minor DG and proposed as candidates for subsequent in vitro and in vivo tests.

Very few studies have focused on the interaction of HS polyphenols with digestive enzymes as their putative molecular targets to explain their effect on obesity. Polyphenols are capable of interacting with proteins through hydrophobic or hydrophilic interactions, leading to the formation of aggregates that can alter and affect its biological activity [64]. The inhibitory effect of HS polyphenols against enzymes implicated in carbohydrate digestion, such as α-amylase activity, and the ability of these compounds to block sugars and starch absorption in an α-amylase-added Caco-2 system, has been reported, which deserves further attention due to is potential implication in weight loss [65,66]. The potential inhibitory activity against pancreatic lipase was also reported by examining the effect of HS extracts on fat absorption-excretion and body weight in rats [67]. This study concluded that animals supplemented with HS polyphenols excreted significant amounts of fat in the feces, mainly palmitic and oleic acids, compared to controls. In addition, the lower weight gain observed in HS-fed animals leads to postulate on an inhibitory effect of pancreatic amylase, in agreement with that proposed by Hansawasdi et al. Thus, continuous administration of HS polyphenols might improve obesity-related metabolic disorders in a similar manner to current digestion inhibitory drugs such as orlistat, which is also associated with triglyceride reductions and adiponectin activation [68]. Although these studies considered hibiscus acid as the main component responsible for the enzymatic inhibition (Figure 1), other recently identified HS compounds could also be responsible by inhibiting pancreatic lipase and/or α-glucosidase. Unfortunately, little is known regarding the molecular mechanisms implicated in the inhibition of these or other digestive enzymes by phenolic compounds. A plausible hypothesis could be that these compounds interact directly with the catalytic site of the enzymes, especially if the site is hydrophobic. In our laboratory, we performed molecular docking experiments of a library containing all the compounds identified in HS extracts (Figure 1) against the catalytic binding sites of different enzymes present in the digestive tract. To this end, the crystal structures of pancreatic lipase and glucosidase enzymes were selected to determine whether HS polyphenols can bind these enzymes with sufficient affinity to be considered as inhibitors. Molecular docking can predict the structure of a receptor-ligand complex and calculates a theoretical Gibbs free-energy variation (ΔG, kcal/mol) for different poses of each ligand. This parameter reflects the intensity and number of atomic interactions between the amino acids of the binding site in the protein and the ligand [69,70]. Figure 2 depicts the Gibbs free-energy variation values calculated through molecular coupling experiments obtained using a library of compounds present in the HS (Figure 1) against the catalytic site of various intestinal glucosidase enzymes (Figure 2A) as well as against two ligand binding sites of the enzymatic complex of the pancreatic triacylglycerol lipase/colipase (Figure 2B). As it can be observed in Figure 2A, the majority of the compounds exhibited lower or similar ΔG values to compounds such as agarbose or naringenin [71], which are known inhibitors of intestinal glycosidases. Therefore, flavonols such as quercetin or kaempferol and their metabolites (Figure 1), which have been identified in plasma of treated animal models, could behave as competitive inhibitors of these glucosidases. Likewise, some phenolic acids or anthocyanins may act as putative ligands of intestinal glucosidase enzymes due to their reasonably low ΔG values. Similarly, the free energy variations for the binding of the HS compounds to the two sites in the lipase/colipase complex are shown in Figure 2B and compared to the drug inhibitor orlistat. As mentioned in the case of intestinal glucosidases, the majority of the HS compounds exhibited lower ΔG values than this drug, and therefore it is plausible to consider these compounds as inhibitors of this enzymatic complex, which could account for the decreased absorption of fatty acids observed in animal models using HS extract, especially for flavonols derivatives. Our computational results based on free-energy variations suggest that these molecules have the potential to inhibit digestive enzymes. Nevertheless, further in vitro and in vivo studies will be required to substantiate whether this in silico approach is an appropriate tool for the identification of new potential inhibitors of digestive enzymes.

## 6. Molecular Effects of *Hibiscus sabdariffa* Polyphenols

### 6.1. Effect on Redox Homeostasis

**Figure 3:**
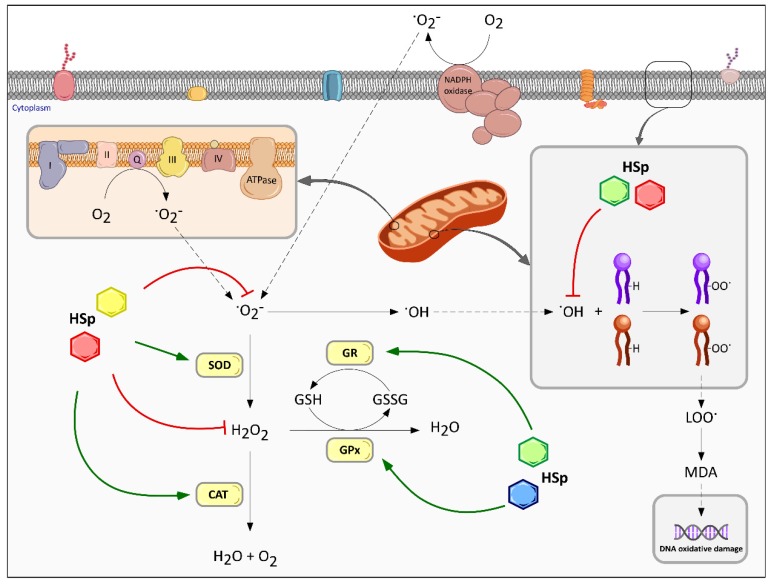
Multiple effects of HS polyphenols on intracellular redox homeostasis. The antioxidant abilities of HS polyphenols (HSp), represented by colored hexagons, act directly or indirectly upon intracellular ROS generation in oxidative status. HSp may directly react with ROS and free radical intermediates by halting the chain reaction, thereby stopping the ROS-induced damage. HSp antioxidant activity blocks intracellular ROS generation in hydrophilic environments and also the generation of lipoperoxy radicals, which are major responsible for DNA oxidative damage. Alternatively, HSp may act indirectly by up-regulating antioxidant enzymes expression or protein activation. SOD, superoxide dismutase; CAT, catalase; GPx, glutathione peroxidase; GR, glutathione reductase. Different colors of HSp symbols indicate different families or structural moieties of polyphenols’ metabolites.

The antioxidant capabilities of plant polyphenols have been well established by many in vitro and in vivo studies, demonstrating a clear correlation with health [16]. To this end, botanical polyphenols could be used as possible therapeutic sources to treat obesity. Oxidative stress is implicated in the development of many chronic conditions, including obesity. This is due to an imbalance between excess reactive oxygen species (ROS) and the inability of the intracellular defense system to efficiently eliminate these oxidative agents. This dysregulation leads to the oxidation and damage of macromolecules such as carbohydrates, lipids, proteins and nucleic acids, which finally cause organelle and cell dysfunction and contributes to the progression of the pathology [72]. ROS are a set of unstable molecules and free radicals derived from molecular oxygen (O_2_) and are mainly generated in the oxidative respiratory chain of the mitochondria. Superoxide anion (·O_2_^−^) is generally the precursor of the majority of the ROS produced, and can lead to the formation of hydrogen peroxide (H_2_O_2_), and consequently hydroxyl radical (OH·) by Fenton’s reaction. Oxidative damage is generally prevented through the release of intercellular and intracellular antioxidant enzymes such as superoxide dismutase (SOD), catalase (CAT), glutathione peroxidase (GPx) and glutathione reductase (GR), which act as scavengers for the different ROS (Figure 3) [73]. Additionally, ·O_2_^-^ and H_2_O_2_ can also be generated by NADPH oxidase, a membrane-bound enzymatic complex, which plays an important role in cellular proliferation, serotonin biosynthesis, endothelial signaling, regulation of renal functions, and the immune response against microorganisms, although its overexpression is associated with various neurological diseases and cancers [74].

**Figure 4:**
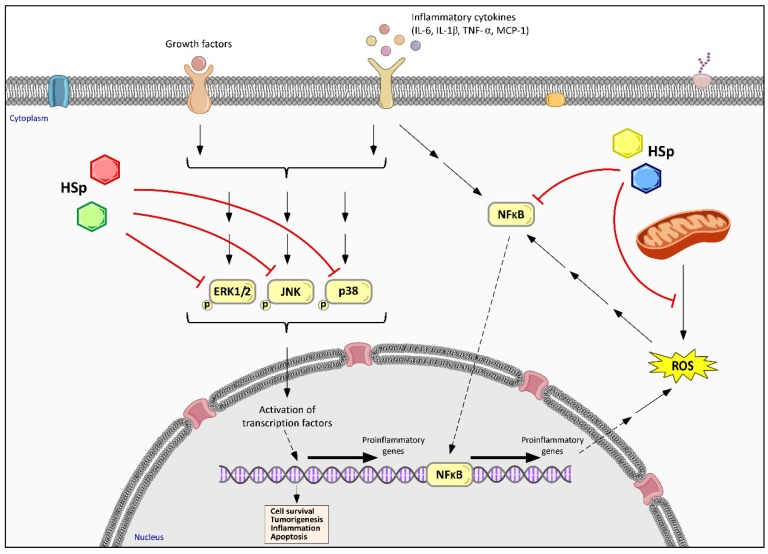
Effects of HS polyphenols on inflammation-related and MAPK pathways. Cellular inhibitory effect of HS polyphenols (HSp), represented by colored hexagons, is associated with a decreased phosphorylation of extracellular signal-regulated protein kinases 1 and 2 (ERK1/2) and c-Jun *N*-terminal kinases (JNK), as well as p38 kinase. HS polyphenols also inhibit inflammation by down-regulating NF-κB pathway and ROS generation, revealing that inflammation, oxidative stress-related pathways and mitochondrial function are closely related and interdependent processes in the HSp effects. Different colors of HSp symbols indicate different families or structural moieties of polyphenols’ metabolites.

A correlation exists between obesity and oxidative stress, as a chronic inflammatory process has been detected in the adipose tissue of obese experimental animal models as well as in humans, along with an increased NADPH oxidase expression and decrease in antioxidant enzymes [75]. Other studies have reported that high levels of fatty acids and glucose increase intracellular ROS generation in adipocyte cell cultures [20,75,76]. According to these reports, adipocytes in obese individuals undergo hypertrophy as a result of an excess caloric intake and a low metabolic rate. Consequently, the increased expression of NADPH oxidase, exacerbated fatty acid oxidation in the mitochondria, and decreased expression of SOD, CAT and GPx lead to an excessive production of ROS. ROS also function as mediators for the activation of nuclear factor-κB (NF-κB) and mitogen-activated protein kinase (MAPK), contributing to the dysregulation of the expression of inflammatory adipokines and a low-grade but chronic state of inflammation (Figure 4) [77].

Various studies have indicated a possible role for HS polyphenols in regulating excess ROS in obesity-related disorders. For example, HS aqueous extracts have shown a higher capability to scavenge peroxyl radicals in water environments than in lipophilic systems, as well as a stronger metal-reducing effect than olive leaf extract [18]. The antioxidant properties of HS polyphenols have also been measured in the plasma of rats after an acute ingestion of a polyphenol-enriched HS extract [19]. In a later study, a correlation between the presence of phenolic acids in plasma at shorter times and its antioxidant effect through ferric ion reduction and superoxide scavenging was reported. Furthermore, the presence of flavonol glucuronides (quercetin and kaempferol) in plasma samples at longer times correlated with an inhibitory effect on lipid peroxidation. Furthermore, a polyphenol-enriched HS extract exerted a higher effect on inhibiting intracellular ROS formation than an aqueous HS extract in a culture of hypertrophied adipocytes [20]. All these data suggest that finding a suitable combination of bioactive HS polyphenols could represent an opportunity to ameliorate obesity-associated oxidative stress.

A correlation between the total phenolic content of HS extracts and their antioxidant properties has been reported [20,78]. Therefore, it must be presumed that the antioxidant properties of the extracts lie on the polyphenol´s abilities (phenolic acids, flavonols and anthocyanins) to scavenge free radicals. This effect has been observed in several oxidative damage models, in both cells and experimental animals. The organic extracts from HS exhibited strong protective properties against malondialdehyde formation and cellular lysis in tert-butyl hydroperoxide-induced oxidative damage in rat primary hepatocytes, suggesting a protective effect against cytotoxicity and genotoxicity [79]. HS anthocyanin extracts have been shown in vitro to scavenge O_2_^-^ radicals and H_2_O_2_, completely attenuate the CCl(4)-mediated decrease in antioxidant enzymes, as well as activate phase II drug detoxification enzymes in CCl(4)-induced oxidative damage of rat livers [80]. Furthermore, the antimutagenic activity and free radical scavenging effects on active oxygen species and lipid peroxidation of HS organic extracts (chloroform and ethyl acetate) has been reported using an oxidative stress-induced rat model, besides revealing a strong ability to scavenge ·O_2_^-^ and OH· radicals and H_2_O_2_ in vitro [81]. These results point to a strong scavenging ability of HS polyphenols at different stages by eliminating ·O_2_^-^, H_2_O_2_ and OH·, which may take place either by simple hydrogen donation or by preventing antioxidant enzyme degradation. Furthermore, the capacity of HS polyphenols to inhibit lipid peroxidation evidenced in different models may be related to their ability to scavenge OH· radicals, which are the major culprits for the generation of lipoperoxyl radicals (ROO·) (Figure 3).

As mentioned above, polyphenols from HS may not only exert their antioxidant effect by directly scavenging ROS, but also by modulating their effect and induce the expression of other antioxidant enzymes. Essa et al. [82] reported that HS extracts increased the levels of SOD, CAT and GPx, and reduce glutathione (GSH) in brain tissues of hyperammonemic rats. In addition, several studies in animal models of metabolic syndrome, diabetes or hyperlipidemia have reported an increase in the expression of SOD, CAT, GPx and GR (Figure 3) in several tissues, such as the kidney, liver and heart [83,84,85,86,87]. These studies support the hypothesis that HS extracts are capable of ameliorating oxidative stress in metabolic diseases.

### 6.2. Hibiscus sabdariffa Effects on Inflammatory and Immune Response

Adipose tissue represents not only a metabolic but also an important endocrine organ. Adipocytes are the main constituent cells in this tissue, and their primary function is to store energy as fat. Nevertheless, adipocytes also play an important role in the secretion of a large number of hormones and cytokines (adipokines) that regulate processes such as lipid metabolism, glucose homeostasis, insulin sensitivity, inflammation, blood pressure or angiogenesis. In addition to adipocytes, other cells such as fibroblasts, pre-adipocytes, vascular endothelial cells and immune cells are also present in the adipose tissue.

In an obese individual, the adipocytes are hypertrophic, leading to molecular and cellular disturbances that can affect their functionality [88]. Accordingly, the secretion of pro-inflammatory adipokines increases in this tissue, including interleukin-6 (IL-6), tumor necrosis factor-alpha (TNF-α), monocyte chemoattractant protein-1 (MCP-1) and vascular cell adhesion molecule-1 (VCAM-1) (Figure 4). This pro-inflammatory phenotype leads to a low-grade inflammation systemic condition including macrophage infiltration in adipose tissue [88,89]. Furthermore, lipid accumulation in other organs such as the muscle, pancreas or liver can induce inflammation and insulin resistance, as well as develop metabolic diseases such as liver steatosis, atherosclerosis or type 2 diabetes [1,90,91]. On the other hand, chronic inflammation and oxidative stress are closely related and interdependent processes that contribute to the pathogenesis of obesity-associated diseases [92].

The anti-inflammatory activity of HS polyphenols has been previously reported in cell and animal models and humans. For example, in the cell model of hypertrophied 3T3-L1 adipocytes, both aqueous and polyphenol-enriched HS extracts inhibited the secretion of eight pro-inflammatory adipokines [20].

Ameliorating the oxidative status might be one of the strategies by which HS polyphenols exert their anti-inflammatory effects, since oxidation and inflammation are closely associated. In this regard, the HS polyphenolic extract has been found to inhibit xanthine oxidase activity in vitro, and decrease nitrite and prostaglandin E2 secretions in LPS-induced cells. Moreover, HS polyphenols also inhibited inflammation by down-regulating cyclooxygenase-2 (COX-2) and inhibiting the activation of c-Jun *N*-terminal kinase (JNK) and p38 kinase, postulating a relationship between HS polyphenols, oxidative stress and suppression of nuclear factor-κB (NF-kB) translocation in a lipopolysaccharide-induced inflammation rat model (Figure 4) [93].

The production of MCP-1 in monocytes/macrophages can be mediated through the activation of extracellular signal-regulated kinases 1/2 (ERK1/2) and JNK as well as the NF-κB pathway [94]. NF-κB is a complex protein capable of regulating the transcription of several genes related to the inflammatory process. HS extracts have been demonstrated to inhibit the expression of NF-κB, as well as decrease the levels of TNF-α, IL-6 and IFN-γ, indicating a hepatoprotective effect in rats with thioacetamide (TAA)-induced hepatotoxicity [49] (Figure 4). Furthermore, oral consumption of an aqueous HS extract in healthy humans has been shown to decrease the plasma levels of MCP-1, suggesting that such an effect is not due to the antioxidant activity itself, but rather the inhibition of inflammatory and metabolic pathways [17].

Since oxidative stress, inflammation and hypertension are also closely linked, the relationship between HS consumption and blood pressure has been investigated by several authors. Joven et al. demonstrated that HS extracts lowered blood pressure in human patients with metabolic syndrome and improved the endothelial function in a rat model. In this study, the consumption of 125 mg/kg/day of HS polyphenols by human patients for four weeks decreased the majority of the inflammatory and oxidative stress biomarkers analyzed, as well as increased the anti-inflammatory hormone adiponectin [22]. The same study revealed that HS extracts reduced the blood pressure in rats, while also inhibiting TNF-α-induced cytokine secretion in rat endothelial cells, suggesting a possible transcriptional down-regulation of glyceraldehyde 3-phosphate dehydrogenase (GAPDH). Furthermore, a reduction of NF-κB and ROS generation and increased nitric oxide synthase (eNOS) and nitric oxide (NO) was observed in the same cells. The results of this study suggest that the hypotensive effect of HS extracts observed in both humans and rats might be mediated by antioxidant, anti-inflammatory and endothelium-dependent mechanisms, in agreement with Hopkins et al. [95]. However, this is in contrast with other studies that have attempted to explain the antihypertensive effectiveness of this extract only through the inhibition of the angiotensin I-converting enzyme [96,97]. In this regard, a recent meta-analysis study on randomized controlled trials using HS confirmed the potential effectiveness, reducing both systolic and diastolic pressure and considered the combination of HS supplements and antihypertensive medications to optimize the antihypertensive therapy [38].

All this evidence leads to the postulation that the anti-inflammatory effects of HS polyphenols are closely related to the modulation of oxidative stress-related pathways. Nevertheless, it must be assumed that the complex mixture of compounds contained in the polyphenolic HS extract may interact with a wide variety of molecular targets directly related to inflammatory processes; therefore, the modulation of gene expression and/or protein activity in these pathways should also be considered. Anyway, the inhibition of MAPK and NF-kB pathways seem to be the major mechanisms of action of HS polyphenols (Figure 4). Thus, regulation of the inflammatory processes in obesity through HS extract consumption could be an interesting approach to ameliorate and/or prevent other chronic inflammatory diseases related to metabolic syndrome such as atherosclerosis, steatohepatitis or cardiovascular diseases.

### 6.3. Modulation of Energy Metabolism and Lipid Management by Hibiscus sabdariffa Polyphenols

Polyphenols comprise of a number of molecular scaffolds with an enormous variety of substitutions with different moieties, either through intermolecular interactions or by polymerization. Therefore, it seems reasonable to consider that such a structural diversity confers the possibility of generating a limitless number of pharmacological compounds for various targets. In this sense, complex polyphenolic mixtures may have a multi-targeted mechanism of action, as reported [10]. As previously commented the different chemical structures contained in the HS extract (Figure 1) have a strong antioxidant capability and protect against chronic inflammation, which have been positively correlated to obesity-related metabolic disorders [48,98]. Nevertheless, these pathologies are complex conditions that require a multifaceted approach, including effects on the inflammatory network, antioxidant status and energy metabolism. Recently, the relationship between polyphenols interactions and energy metabolism modulation has been reported, suggesting that the consumption of plant-derived polyphenols can change lipid and energy metabolism and may facilitate weight loss and prevent weight gain [15]. 

The ability of HS polyphenols to modulate energy metabolism in order to explain the potential beneficial effects on lipid management and weight loss has been studied by several authors in cell and animal models. The polyphenol-enriched HS extract presented a higher efficiency in inhibiting intracellular triglyceride accumulation than aqueous HS extract that contained higher polysaccharide content in a culture of hypertrophied adipocytes [20]. This observation points to the polyphenols as the candidate molecules responsible for the lower lipid accumulation, indicating that it may be a suitable strategy to improve obesity-associated disturbances. Several studies have proven the ability of HS polyphenols to help reduce body weight through inhibition of fat accumulation, while also improving glucose tolerance and normalize the glycemic index in obese mice models [23,99].

**Figure 5:**
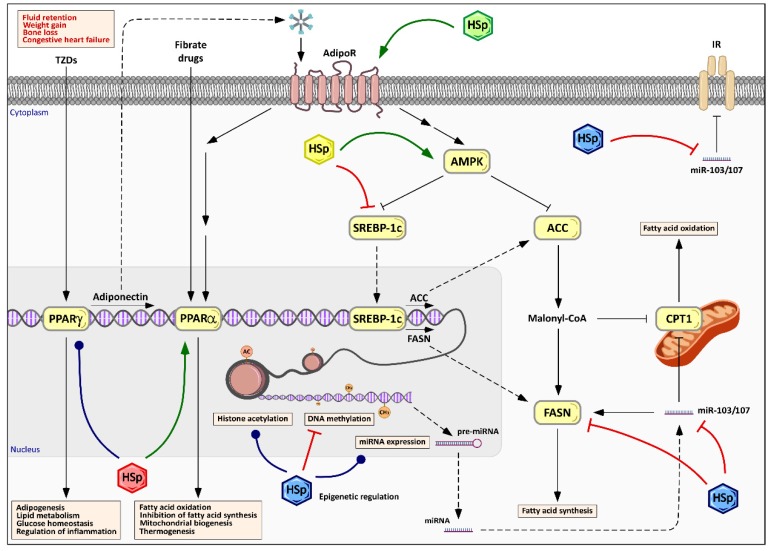
Pleiotropic effects of HS polyphenols on cellular energy metabolism and lipid and glucose homeostasis. HS polyphenols (HSp), represented by colored hexagons, stimulate AMP-activated protein kinase and, consequently, inhibit downstream targets, such as acetyl-CoA carboxylase (ACC) and fatty acid synthase (FASN), decreasing the fatty acids synthesis and stimulating lipolysis. Alternatively, HSp action, either directly or through AMPK, promotes downregulation of FASN by the inhibition of the sterol regulatory element-binding protein-1c (SREBP-1c). The modulation of peroxisome proliferator-activated receptors (PPAR) expression and/or activity, and the subsequent adiponectin expression increase, in response to HSp improves lipid and glucose homeostasis and mitochondrial biogenesis. The pleiotropic effects of HSp also include primary control mechanisms such as epigenetic modifications through histone acetylation, DNA methylation and miRNA expression. Different colors of HSp symbols indicate different families or structural moieties of polyphenols’ metabolites.

Its effect on weight loss has prompted hibiscus extract to be examined for its potential effect on lipid profiles. Several studies have indicated that HS extracts have a lipid lowering activity, which could prevent cardiovascular disease through lipid modification [100]. Fernández-Arroyo et al. [18] tested HS polyphenols on low density lipoprotein receptor deficient mice (LDLr^−/−^) under a hypercaloric diet (20% fat and 0.25% cholesterol, *w/w*). A strong correlation between HS consumption and the ability to decrease serum triglyceride concentration was reported in this study. Several studies using hyperlipidemic rat models together with continuous cholesterol feeding for four weeks resulted in a significant reduction of serum cholesterol, triglycerides, LDL and VLDL levels [101,102]. Since the majority of the plasma apolipoproteins, endogenous lipids and lipoproteins are synthetized in the liver, several studies have attempted to establish a link between the hypolipidemic ability of HS and its putative mechanism on the liver. Yang et al. [24] examined the effect of HS on liver fat metabolism and revealed that HS polyphenols reduced liver damage by promoting lipid clearance. A decrease in the plasma lipid level and hepatocyte lipid content concomitantly with the activation of AMPK, along with an inhibition of fatty acid synthase (FASN) expression, was also detected. A similar effect has been observed in hyperlipidemic mice model, in which the administration of the HS polyphenolic extract prevented fatty liver disease associated with changes in lipid and glucose metabolism, as well as AMPK activation and decreased expression of lipogenic genes such as FAS and SREBP-1c [21] (Figure 5).

It is known that the activation of AMPK, a master regulator of energy metabolism, and the subsequent inhibition of acetyl-CoA carboxylase play a crucial role in fatty acid oxidation by regulating mitochondrial availability of fatty acids [103] (Figure 5). Both AMPK and PPARs have been shown to play an important role in the pathogenesis of both alcoholic liver disease and non-alcoholic fatty liver disease, in which fatty acid oxidation is impaired. Administration of AMPK or PPAR-α activators have shown to be effective in ameliorating both diseases [104]. Polyphenols have also been shown to upregulate PPAR-γ-mediated adiponectin expression, along with AMPK activation [12]. Therefore, regulating the complex network of energy metabolism and mitochondrial function may require a multifaceted approach, including AMPK and PPARs, which can be addressed by the pleiotropic character of plant-derived polyphenols. 

HS polyphenols have been proven to regulate AMPK as well as several transcription factors related to lipid and glucose homeostasis, such as PPARs and SREBP-1c in hyperlipidemic mice model [21,23] (Figure 5). Growing evidence indicates that these transcription factors are critical regulators of hepatic lipid metabolism, stimulating the expression of several enzymes implicated in liver fatty-acid synthesis, glucose transport and gluconeogenesis [23]. Consistent with this hypothesis, a recently discovered low molecular weight compound acting as an adiponectin receptor (AdipoR) agonist, AdipoRon, has been proposed as a possible molecular tool to improve insulin resistance, by mediating the activation of AMPK and PPAR-pathways, mimicking the effect of adiponectin [3]. Thiazolidinediones are oral medications for type 2 diabetes that function as synthetic ligands and potent agonists of PPAR-γ, being highly effective in reducing glucose levels and improving insulin sensitivity. However, the administration of thiazolidineones is associated with the occurrence of severe side effects such as fluid retention, weight gain, cardiac hypertrophy, bone fractures and hepatotoxicity (Figure 5) [6]. The use of polyphenols as mild PPAR-γ agonists based on selective cofactor-receptor interactions, while avoiding the side effects observed in synthetic agonists, has been postulated as an opportunity for the management of obesity [69]. In fact, some polyphenols such as resveratrol [105] or scutellarin [106] have shown experimentally the capability to modulate PPAR-γ. Moreover, extensive effort has been recently made on the design of alternative synthetic PPAR modulators, such as metaglidasen, that are devoid of the typical side effects observed with thiazolidinedione anti-diabetic agents [107]. The ability of HS polyphenolic extracts to activate AMPK has also been associated with the activation of PPAR-α, which triggers FASN inhibition, stimulating fatty acid oxidation and antioxidant enzyme expression, thereby reducing lipid content and oxidative stress, which enhance mitochondrial metabolism and lipid management [21] (Figure 5). HS polyphenols has also been correlated with a significant increase in serum adiponectin and decrease in serum leptin, which can revert overweight-induced metabolic alterations [22].

### 6.4. Epigenetic Effects of Hibiscus sabdariffa Polyphenols

The majority of the studies on the effects of HS and other plant polyphenols have focused on specific molecular targets in order to explain their molecular mechanism in the amelioration of obesity-related pathologies. As such, several cellular pathways, transcription factors and enzymatic activities seem to be modulated by these compounds. This mode of action suggests the existence of a primary control mechanism, which appears to be in line with the pleiotropic character and structural diversity of these compounds. Recent studies have shown that plant polyphenols are capable of exerting an epigenetic control. Epigenetics include a number of extra-genetic processes such as DNA methylation, post-translational histone modifications and non-coding RNA mediated gene silencing, all of which can alter gene expression, but do not involve DNA sequence changes and can be modified by environmental stimuli [108]. Plant derived polyphenols, such as curcumin, catechins, resveratrol or some flavonols, can interact with various enzymes and important epigenetic modifiers, such as histone acetyltransferases, histone deacetylases, DNA methyltransferases, kinases and miRNA [109,110]. 

Several authors have reported that polyphenols can reverse the altered epigenetic modifications observed in metabolic disorders by changing DNA methylation and histone modifications [111]. For example, polyphenols are effective histone deacetylases inhibitors and thus can be used to reverse the reduced histone acetylation associated with neurodegeneration [112,113]. Quercetin, a fairly abundant flavonol in the polyphenolic fraction of HS extracts, inhibited the histone acetyltransferase activity in the promoter region of genes associated with inflammation [114]. Currently, the epigenetic regulation of energy balance and adipose tissue biology has been proposed by modulating the expression of certain microRNAs [115]. HS polyphenols have been shown to be capable of regulating microRNA expression in hyperlipidemic mice with LDL receptor deficiency [21]. The chronic oral administration of HS polyphenols in mice under a fat-enriched diet reverted the changes observed in non-specific microRNAs miR103/107 concomitantly with the prevention of diet-induced fatty liver disease and changes in lipid and glucose metabolism (Figure 5). Recently, the ability of HS polyphenols to regulate gene expression through the epigenome has been confirmed [116]. Research is currently focused on elucidating whether HS polyphenols and their metabolites are capable of regulating epigenetic modifications through DNA methylation, histone post-translational modifications of miRNA expression modulation.

## 7. Virtual Screening of *Hibiscus sabdariffa* Polyphenols on Selected Protein Targets

**Figure 6:**
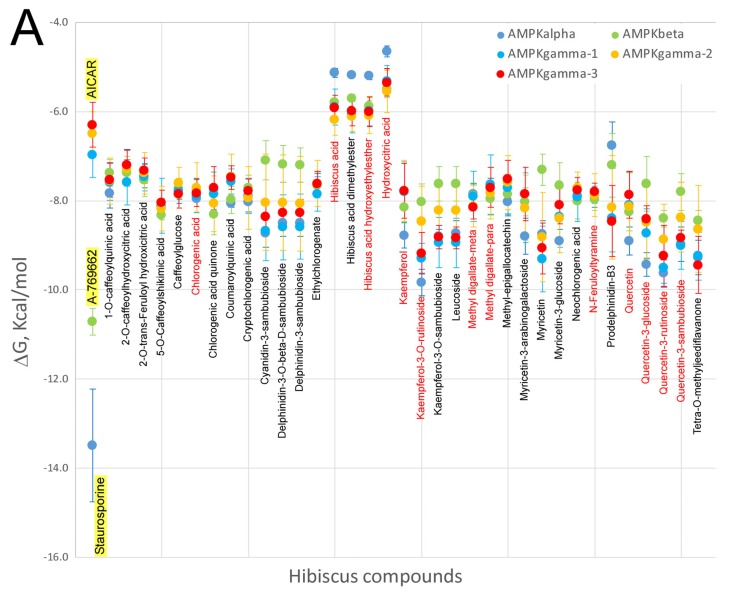

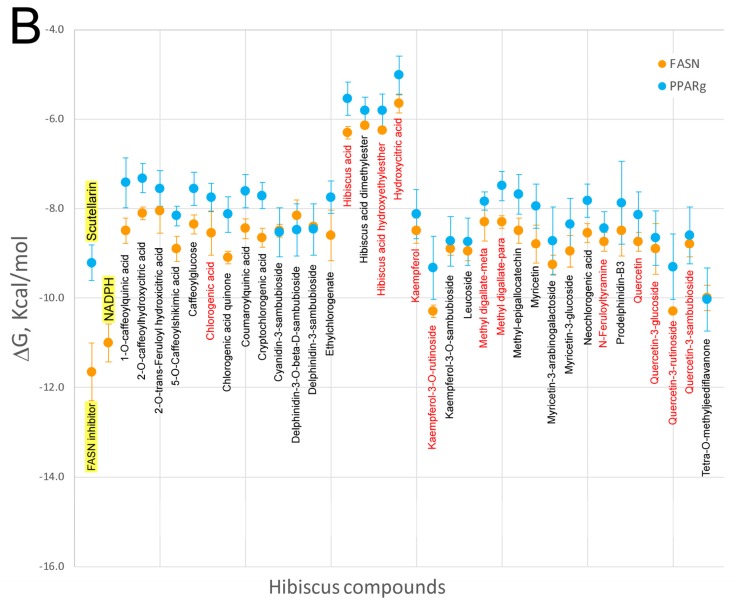
Comparison of the free energy variation (ΔG, kcal/mol) of the *Hibiscus sabdariffa* bioactive compounds and X-ray co-crystalized ligands or known modulators (first name of each panel and yellow background) against the main binding sites of: the catalytic binding site of the three AMPK subunits obtained (A); and the catalytic binding site for fatty acid synthase and binding cavity of PPAR-gamma nuclear receptor (B), by molecular docking. The common name of each compound is included under mean and standard deviation plotted in each all four panel. Red color names indicate that these compounds have been identified as plasma metabolites in the bibliography. Virtual screening process was performed as mentioned in Figure 2.

Many studies have indicated that HS polyphenols are capable of targeting several proteins that are implicated in obesity-associated metabolic disorders, i.e. AMPK, PPAR and FASN. In order to elucidate on the possibility that HS polyphenols may exert a direct effect on these proteins, virtual screening using a library of chemical structures was assessed (Figure 1). The abundant high resolution structural information of these proteins, especially for PPAR and AMPK, allowed us to perform molecular docking approaches in order to try to understand if HS compounds, especially those detected in blood plasma as metabolites, could interact with these proteins at their catalytic or regulatory sites. The results are shown in Figure 6A (AMPK kinase) and Figure 6B (fatty acid synthase and PPARgamma). AMPK is a cellular energy state-sensitive kinase that is activated under deficient energy states due to lack of nutrients or hypoxia [117]. In addition, it can be activated when certain proteins are phosphorylated, promoting the inactivation of energy-consuming pathways and activating the catabolism of fatty acids and other fuels [118]. The AMPK gamma subunit has three binding sites for AMP/ADP or other modulators, which activate and maintain the phosphorylation of threonine 172 within the activation loop of the catalytic alpha subunit kinase. Here, we have performed molecular docking with the x-ray co-crystallographic ligands and AMPK subunits on its binding sites as a control of the most adequate ΔG in each binding site. X-ray value for AICAR as ligand on the three sites of the AMPK gamma subunit presented a ΔG ≈ −6.5 ± 0.5 kcal/mol when docked over its respective binding sites for all the available PDBs: 2UV4, 2UV5, 2UV6, 2UV7, 4CFE, 4CFF, 4RER, 4REW, and 4ZHX [118]. As can be observed in Figure 6A, with the exception of hibiscus acid and its derivatives, all HS compounds, especially those present in rat blood plasma as metabolites (its most commonly-used name is indicated in red text, Figure 1), show ΔGs decrease up to 2 kcal/mol compared to the controls included in the graph, AICAR in this case. Therefore, these results seem to indicate that most HS compounds excluding organic acids might act as potential activators for the AMPK gamma subunit. At the interface of the interaction between the alpha catalytic and beta regulatory subunits of AMPK, crystallographic data show an additional regulatory site for this enzyme. Molecular docking of the A-769662 activator against this regulatory site presents a ΔG ≈ −10.72 ± 0.30 kcal/mol [118]. Our docking data against this regulatory site shows that HS compounds exhibit ΔG values 2 kcal/mol greater than A-769662 inhibitor; therefore, we would not expect them to exert any regulatory role on this binding site. When the ΔG values of the HS compounds against the catalytic site of the AMPK alpha subunit were compared with the ΔG value (−13.5 ± 1.3 kcal/mol) of staurosporine, which is an ATP-competitive kinase inhibitor with a high affinity but little selectivity, it was observed that none of the HS compounds presented such low ΔGs values. As such, they would not behave as competitive AMPK inhibitors. Finally, Figure 6B compares the ΔGs of HS compounds with those of known PPARgamma and FASN modulators or inhibitors. Only certain HS compounds (Kaempferol-3-O-rutinoside, quercetin-3-rutinoside or tetra-O-methyljeediflavanone) presented a ΔG comparable to that of scutellarine, a flavone for which there is experimental evidence [106] of its ability to bind to PPARgamma and thus could activate this nuclear receptor. Interestingly, these same three compounds, and also quercetin 3,7-diglucuronide showed a ΔG lower than −10 kcal/mol for FASN docking, which is comparable to that detected in the GSK2194069 FASN inhibitor [119], making us to think that they could also behave as inhibitors of this enzyme implicated in fatty acid biosynthesis. Taken together, the data derived from the molecular docking experiments of HS compounds *versus* several binding sites to different proteins involved in energy metabolism, seem to indicate that several compounds extracted from this plant might exert a direct effect on these proteins. Of course, further studies, both in vitro and in vivo, will be required to corroborate this hypothesis and understand the mechanisms by which these compounds exert their effect on the corresponding proteins.

## 8. Potential Molecular Mechanism of *Hibiscus sabdariffa* in Obesity: A Global Analysis 

Obesity-related metabolic disorders are characterized by early cellular events and dysregulation of normal cellular homeostasis. In this sense, obesity must be understood as a multidimensional disease, ranging from adipocyte hypertrophy to the appearance of symptoms related to metabolic alterations. HS derived polyphenols could approach the therapy of this pathology from a global perspective, taking advantage of the pleiotropic character of these compounds. Several altered metabolic biomarkers and proteins have been validated as a prequel in the development of bioenergy alterations. Thus, crucial proteins such as PPAR, FASN, lipase, adiponectin, leptin, MCP-1, AMPK, NF-kB and SOD, among others, have been proposed by several authors as specific targets in the treatment of obesity-associated metabolic disorders. In this scenario, HS polyphenols seem to interact with all the above-mentioned targets. In addition, their antioxidant capacity may explain many of the benefits granted to this plant.

Among the putative processes involved in the molecular effects of HS polyphenols on obesity are: modulation of the signaling and energy metabolism pathways, regulation of redox homeostasis and inflammation, restoration of mitochondrial functionality and epigenetic machinery regulation. The last process may probably play a key role on the other aspects.

The extensive literature on the multiple beneficial effects that HS extract exert on obesity-related diseases, as well as its putative mechanism of action, provides sufficient evidence to conclude that the use of this extract could become an adjuvant in these pathologies. Despite the fact that the majority of the cellular and animal effects observed with HS polyphenols have been corroborated in various human trials, further studies are required to define the adequate dosage for therapy as well as validate its use in the management of obesity [116]. Therefore, future research should pay attention to identify the cellular metabolites of HS acting as final effectors through in vivo experiments and powerful analytical platforms.

In this regard, a recent study on the acute multifunctional effects of HS polyphenols in humans have used a combined transcriptomics and metabolomics approach to determine the modifications in the expression of relevant genes through network-based methods [53]. Their results suggest that these polyphenols exert simultaneous effects in mitochondrial function, energy homeostasis, oxidative stress and inflammation, as well as cardiovascular-protective effects, acting upon multiple targets: proteins, hormones and physiological peptides. These findings support the hypothesis that polyphenols are extremely bioactive in humans, and the observed effects are the result of numerous beneficial and synergistic interactions, through the modulation of multiple metabolic pathways and epigenetic regulation [53].

Although the potential pharmacological and therapeutic superiority of the combination of polyphenols in HS extract with respect to their individual components for obesity-related pathologies seems to be demonstrated, several issues related to bioavailability, dosage and safety need to be addressed before its use in human nutrition. First, human equivalent dose must be established from animal trials to assure its efficacy in metabolic disorders. Second, the stability and absorption of these formulations has to be explored to maximize the efficacy of these formulations in human nutrition [10,20]. Finally, although the combination of polyphenols from HS appears to be safe from its traditional use in several cultures, the subchronic administration of these mixtures has to be assayed for toxicity due to the potential polyphenol toxicity itself on a long-term basis or to the presence of some contaminants in plants (mycotoxins, pesticides or metals) [120].

When explaining the multi-targeted effects that plant-derived polyphenols have on proteins targets, an intriguing question arises: are we facing a large variety of molecules bearing a few moieties able to interact with many protein targets, or are the responsible compounds just a few simple and yet to be identified metabolites derived from many polyphenolic structures and acting at primary control epigenetic mechanisms?

Nevertheless, the use of partial or biased approaches focused on specific targets will probably fail to elucidate the precise mechanism of the polyphenols. On the contrary, to fully understand the health and multi-target effects that polyphenols have on obesity and human health, it is necessary to integrate transcriptomic, proteomic and targeted metabolomic approaches, with the support of virtual screening techniques of metabolites on selected protein targets. Lastly, the use of epigenetic approaches will be required to understand how these effects are regulated, as well as determine the most adequate biomarkers for disease diagnostics and prognostics, as well as for treatment. Only with this global approach we will be able to succeed in the design of polyphenolic mixtures sufficiently effective to ameliorate obesity-related pathologies through nutritional intervention or pharmacological treatment.

## Abbreviations

Hibiscus sabdariffa	HS
low-density lipoprotein	LDL
LDL receptor	LDLr
reactive oxygen species	ROS
Sterol regulatory element-binding proteins	SREBP-1c
AMP-activated protein kinase	AMPK
fatty acid synthase	FASN
peroxisome proliferator-activated receptor	PPAR
superoxide dismutase	SOD
catalase	CAT
glutathione peroxidase	GPx
glutathione reductase	GR
diode array detection	DAD
electrospray	ESI
high-performance liquid chromatography	HPLC
